# Experiencia en Cirugía de Miocardiopatía Hipertrófica Obstructiva en un Centro de Referencia Nacional

**DOI:** 10.47487/apcyccv.v1i1.12

**Published:** 2020-03-30

**Authors:** Víctor Robles, Yemmy Pérez, Josías Ríos

**Affiliations:** 1 Servicio de Cirugía Cardiovascular Adulto - Instituto Nacional Cardiovascular INCOR. Lima, Perú. Servicio de Cirugía Cardiovascular Adulto - Instituto Nacional Cardiovascular INCOR Lima Perú

**Keywords:** miocardiopatía hipertrófica, cirugía, miectomía septal, hypertrophic obstructive cardiomyopathy, surgery, septal myectomy

## Abstract

**Objetivos:**

Identificar las características clínicas, analizar los resultados y mostrar la eficacia del tratamiento quirúrgico de la miocardiopatía hipertrófica obstructiva (MHO), en un hospital de referencia nacional

**Material y métodos:**

Se realizó un estudio descriptivo, retrospectivo, tipo serie de casos, de pacientes operados en el Instituto Nacional Cardiovascular con el diagnóstico de MHO, entre diciembre del 2016 y enero del 2019. Se analizó la evolución posoperatoria de la sintomatología, clase funcional (CF), gradiente del tracto de salida del ventrículo izquierdo (GTSVI) y de la insuficiencia mitral (IM).

**Resultados:**

Se evaluaron 13 casos con MHO sometidos a miectomía septal extendida. Un 31% fueron mujeres y la edad media fue de 39.6 años. Antes del tratamiento quirúrgico 85% se encontraba en CF III-IV, 85% de pacientes presentaba IM severa, el grosor medio del septum interventricular era de 27 mm (rango de 19 a 39 mm) y el GTSVI medio, de 111 mmHg (rango de 60 a 150 mmHg). Luego del tratamiento quirúrgico se observó mejoría de la CF (69% en CF I)y del grado de IM (92% con IM nula o mínima), y reducción de GTSVI media a 16 mmHg (rango de 6 a 35 mmHg). En 7 pacientes (54%) se realizó cirugía simultánea de la válvula mitral.

**Conclusiones:**

Nuestra experiencia en cirugía de MHO es adecuada. El tratamiento simultáneo de los componentes miocárdicos y valvular permite reducir el GTSVI y corregir la IM.

## Abstract (en)

**Objectives:**

To identify the clinical characteristics, analyze the results and show the efficacy of surgical treatment of hypertrophic obstructive cardiomyopathy (HOC), in a national reference institute.

**Methods:**

A descriptive, retrospective, case series of patients with the diagnosis of HOC operated at the National Cardiovascular Institute, was performed between December 2016 and January 2019. We analyzed the postoperative evolution of symptomatology, functional class (FC), left ventricular outflow tract gradient (LVOTG) and mitral regurgitation (MR).

**Results:**

Thirteen cases with HOC undergoing extended septal myectomy were evaluated. 31% were women and the average age was 39.6 years. Before surgical treatment, 85% were in functional class (FC) III-IV, 85% of patients had severe MR, the mean septum thickness was 27 mm (range from 19 to 39 mm), and mean LVOTG was 111 mmHg (range from 60 to 150 mmHg). After surgical treatment we found improvement of the functional class (69% in FC I) and the degree of MR (92% with zero or minimal), and reduction of mean LVOTG to 16 mmHg (range from 6 to 35 mmHg). Simultaneous surgery of the mitral valve was performed in 7 patients (54%).

**Conclusions:**

Our experience in HOC surgery is good. The treatment of both myocardial and valve components allows reducing LVOTG and correcting MR.

Estudios epidemiológicos basados en estudios ecocardiográficos muestran una prevalencia de la miocardiopatía hipertrófica obstructiva (MHO) de 1 caso por 500 personas en la población general, con ligero predominio del sexo masculino.^1^^,^^2^ Etiológicamente, en un 60% de los adolescentes y adultos con MHO la enfermedad tiene un rasgo autosómico dominante, causado por mutaciones en más de 11 genes que codifican los componentes proteicos del sarcómero. ^(^^3^

La fisiopatología de la MHO, está caracterizada por una obstrucción dinámica, de intensidad variable, normalmente localizada en posición subvalvular aórtica, entre el septum interventricular hipertrofiado y la válvula mitral, asociada con frecuencia a un movimiento sistólico anterior (MAS) de la vávula mitral que produce diversos grados de insuficiencia mitral (IM).^1^

El diagnóstico de la MHO se basa en la detección de un aumento del grosor de la pared del ventrículo izquierdo (VI) mediante cualquier modalidad de imagen (ecocardiografía, resonancia magnética, tomografía computarizada). En el adulto se define por un grosor de la pared ≥ de 15 mmm en uno o más segmentos miocárdicos del VI, que no pueda explicarse únicamente por condiciones de carga. ^(^^4^ La obstrucción del tracto de salida del ventrículo izquierdo (OTSVI) se define como un gradiente doppler instantáneo máximo del tracto de salida del ventrículo izquierdo ≥ 30 mmHg, pero el umbral a considerar para el tratamiento invasivo es de ≥ 50 mmHg. ^(^^4^ En la evolución clínica de la MHO, la mayoría no presentan síntomas clínicos y/o eventos adversos relevantes, no requieren tratamiento y cursan con una esperanza de vida normal. Un 5% de los pacientes evolucionan con síntomas persistentes y son candidatos a una terapia invasiva, ya sea con el desfibrilador automático implantable (DAI), ablación miocárdica septal transluminal percutánea o cirugía.^5^

El tratamiento invasivo para reducir la OTSVI se debe considerar en pacientes con un gradiente ≥ 50 mmHg, síntomas de moderados a graves (clase funcional NYHA III - IV), o síncope de esfuerzo recurrente a pesar de recibir tratamiento farmacológico a dosis máximas toleradas.^6^ Por la complejidad del sustrato anatómico que produce la obstrucción en la MHO se describe diferentes estrategias quirúrgicas que se han reportado en los últimos años.^7^^,^^8^ El tratamiento quirúrgico más usual para tratar la OTSVI es la miectomía ventricular (procedimiento de Morrow).^9^ Un grupo de pacientes requiere cirugía mitral concomitante como la sustitución de la válvula mitral, reali-neación, escisión parcial o movilización de los músculos papilares, plicatura o extensión de la valva anterior.^10^ Otra opción quirúrgica es la miectomía septal mínimamente invasiva trans-mitral con reparación y/o reemplazo de la válvula mitral.^11^

El objetivo de este trabajo fue analizar los resultados del tratamiento quirúrgico en pacientes con MHO que recibieron terapia farmacológica máxima sin mejoría clínica, en el Instituto Nacional Cardiovascular INCOR del Perú.

**Tabla 1:** Características basales

Edad	39.6	(± 17.4)
Varones	9	(69)
Antecedente familiar de MHO	11	(85)
Disnea	13	(100)
Angina	7	(54)
Síncope	4	(31)
Portador de DAI	9	(69)

Se reporta medias (desviación estándar) y frecuencias (porcentaje) para variables cuantitativas y categóricas, respectivamente.

MHO: miocardiopatía hipertrófica obstructiva; DAI: defibrilador automático implantable.

## Material y Método

Se realizó un estudio descriptivo, retrospectivo, tipo serie de casos, de pacientes con MHO, intervenidos quirúrgicamente en el Instituto Nacional Cardiovascular - INCOR EsSalud del Perú entre diciembre del 2016 a enero del 2019. La recolección de datos se realizó mediante la revisión de las historias clínicas. Se utilizó para el análisis y elaboración de resultados las siguientes variables: edad, sexo, antecedentes familiares, estudios diagnósticos realizados en el preoperatorio, signos y síntomas preoperatorios y postoperatorios, clase funcional preoperatorio y postoperatorio, grado de insuficiencia mitral (IM) en el preoperatorio y postoperatorio, fracción de eyección del ventrículo izquierdo (FEVI) preoperatorio, grosor del septum interventricular pre y postoperatoria, gradiente del tracto de salida del ventrículo izquierdo (GTSVI) pre y postoperatorio, técnica quirúrgica realizada, cirugía de la válvula mitral, uso de desfibrilador automático implantable (DAI) y estancia hospitalaria. Las variables numéricas se expresaron en media y desviación estándar, mediana y rango intercuartil y las categóricas en porcentajes. Se analizaron las diferencias entre grupos con la prueba de T de Student para muestras independientes o con la prueba en U de Mann-Whitney para variables continuas y la prueba de chi cuadrado para variables categóricas. Se consideró estadísticamente significativo el valor de p < 0.05. Los datos fueron sometidos al análisis en el programa estadístico STATA 15. 

## Resultados

Entre diciembre del 2016 y enero del 2019 se intervinieron quirúrgicamente 13 pacientes con el diagnóstico de MHO en el Instituto Nacional Cardiovascular INCOR - EsSalud. Once pacientes (85%) presentaban antecedentes familiares de MHO. La edad de los pacientes fluctuó entre los 14 años y los 67 años, y la mayoría de ellos fueron varones (69%). Todos los pacientes tenían terapia farmacológica máxima sin mejoría de la sintomatología y 9 pacientes (69%) eran portadores de DAI. (Tabla 1)

El estudio ecocardiográfico se realizó en el 100% de los pacientes y el cateterismo cardíaco en 46%. Los síntomas más frecuentes de presentación fueron disnea, angina y síncope. El grado de IM fue evaluado en el preoperatorio, intraoperatorio y postoperatorio por medio de ecografía transtorácica y transesofágica. En 12 pacientes se realizó la miectomía septal extendida según la técnica clásica (Morrow) y en 1 paciente se realizó miectomía septal transmitral por mini toracotomía anterolateral derecha videoasistida, cuyos resultados quirúrgicos fueron similares a la técnica clásica. 

## Procedimiento quirúrgico

La cirugía se realizó, en todos los casos, con control de ecocardiografía transesofágica. En 12 pacientes se realizó una esternotomía media, canulación en aorta ascendente y de ambas cavas, heparinización y entrada a circulación extracorpórea, llevando al paciente a una temperatura de 32°C. La parada cardíaca con protección miocárdica se realizó usando cardioplejia sanguínea. Se realizó una aortotomía oblicua dirigida hacia el velo aórtico no coronariano para acceder al septum interventricular. Se separó cuidadosamente los velos aórticos y con un bisturí número 15 se practicó una resección de un fragmento del septum interventricular por medio de dos incisiones paralelas, la primera debajo del nadir del velo coronariano derecho y la segunda (en contra de las agujas del reloj) debajo de la comisura del velo coronariano derecho e izquierdo, respetando 5 mm debajo del anillo valvular aórtico. La resección se prolongó distalmente hasta la base de la implantación de los músculos papilares (miectomía septal extendida). (Tabla 2)

**Tabla 2:** Características del intraoperatorio

Tiempo de pinzamiento de aorta (minutos)	82.6	(± 36.5)
Tiempo de circulación extracorpórea (minutos)	138.2	(± 60)
Plastía de la válvula mitral (pacientes)	3	(23.1)
Reemplazo de la válvula mitral (pacientes)	4	(30.8)
Tiempo en cuidado intensivo (días)	2.9	(± 1.1)

Se reporta medias (desviación estándar) y frecuencias (porcentaje) para variables cuantitativas y categóricas, respectivamente

En 6 pacientes se realizó miectomía aislada (46%) y en 7 pacientes (54%) se realizó cirugía de válvula mitral concomitante (3 valvuloplastias y 4 implantes de prótesis valvular) con abordaje por atrio izquierdo. En el único caso de miectomía septal transmitral mínimamente invasiva, se hizo con canulación arterial y venosa vía femoral, y el abordaje fue por una minitoracotomía anterolateral derecha videoasistida. Al terminar la circulación extracorpórea se realizó en todos los casos el control ecocardiográfico transesofágico para comprobar la reducción del GTSVI y la corrección de la IM.

## Desenlaces

El 85% de los pacientes en el preoperatorio se encontraban en CF III-IV y luego del tratamiento quirúrgico, el 100% de los pacientes sobrevivientes pasaron a CF I-II. El gradiente medio del tracto de salida del VI disminuyó de 111.4 mmHg (preoperatorio) a 15.7 mmHg (postoperatorio). En cuanto a la IM, el 85% de los pacientes presentaba IM severa y en el postoperatorio el 92% tuvo IM leve o nula. (Tabla 3)

Se encontraron dos eventos intrahospitalarios post cirugía: un paciente portador de DAI falleció al decimosegundo día postoperatorio por una arritmia sin respuesta al tratamiento. En otro paciente el control ecocardiográfico al primer día postoperatorio encontró IM severa a pesar de haberse realizado la valvuloplastia mitral por lo que se procedió a una segunda intervención para reemplazo valvular mitral sin complicaciones.

**Tabla 3:** Características ecográficas pre y postoperatorias

	Pre Operatorio		Post Operatorio	
Septum basal	27.1	(± 6.1)	19.3	(± 8.3)
Gradiente en TSVI	111.4	(± 35.9)	15.7	(± 9.8)
Insuficiencia mitral moderada o severa	13	(100)	1	(8.0)

Se reporta medias (desviación estándar) y frecuencias (porcentaje) para variables cuantitativas y categóricas, respectivamente.

TSVI: Tracto de salida de ventrículo izquierdo

## Discusión

La elección del tratamiento para la MHO es diversa y la historia natural, impredecible. Diversos estudios retrospectivos han estimado una mortalidad anual aproximada menor del 1%^2^^,^^12^ y muchas de las muertes son súbitas, presumiblemente causadas por arritmias ventriculares.^1^ Otras complicaciones que pueden ocurrir incluyen fibrilación auricular, endocarditis infecciosa y estadios finales de insuficiencia cardíaca.^13^ Los betabloqueadores y calcioantagonistas pueden dar mejoría sintomática a muchos pacientes con MHO, especialmente en aquellos sin obstrucción u obstrucción latente, pero hay considerables variaciones en la respuesta individual a las drogas.^14^

El manejo depende de la sintomatología del paciente. En los sintomáticos se debe usar betabloqueadores y calcioantagonistas, y como última opción queda el manejo invasivo que se divide en dos modalidades: ablación con alcohol y la miectomía quirúrgica que disminuye el grosor del septum, corrige la OTSVI y la IM. En un 5% de los pacientes la sintomatología es refractaria al tratamiento farmacológico y se debe considerar terapia invasiva.^6^^,^^15^

Para los pacientes con MHO y OTSVI, el tratamiento quirúrgico da mejoría sustancial de los síntomas y puede mejorar el pronóstico.^16^^,^^17^ La miectomía septal transaórtica como procedimiento estándar fue desarrollado por Morrow et al,^9^ es excelente en liberar la OTSVI y es altamente reproducible. Otras alternativas quirúrgicas como la miectomía septal mínimamente invasiva se proponen seguras y requieren experiencia e instrumental especializado.^11^^,^^18^

La miectomía septal extendida incrementa el diámetro del tracto de salida del ventrículo izquierdo con la disminución del gradiente y la velocidad de flujo, y corrige la IM funcional. Sin embargo, el tratamiento debe considerarse no solo para el septum hipertrófico, sino también para la válvula mitral y el aparato subvalvular pues la presencia de alteraciones anatómicas de la válvula mitral en pacientes con MHO está bien documentada.^19^^,^^20^ Los velos mitrales son habitualmente más grandes de lo normal y los músculos papilares adoptan una posición más anterior dentro de la cavidad ventricular. En ese sentido, la plicatura transversal,^21^ y la movilización y escisión parcial de los papilares^22^ se describen como técnicas quirúrgicas para el manejo de patología mitral. Estas técnicas deben limitarse a aquellos con MAS, IM severa y velos grandes y redundantes. 

El reemplazo valvular mitral, posiblemente el trata-miento más radical de la MHO para conseguir una mayor reducción de la GTSVI y corregir la IM, debe realizarse resecando toda la válvula y el aparato subvalvular, incluidos los músculos papilares.^23^ Si desaparece el velo anterior no existe posibilidad de MAS. Sin embargo, esta debe ser una alternativa en casos seleccionados, debido a las complicaciones propias de las prótesis.^24^

En nuestra experiencia, la cirugía logró una reducción significativa del GTSVI, de la IM y una mejoría sintomática. Las cifras del GTSVI mostradas se encuentran dentro de los límites de otros estudios internacionales (que oscilan entre 4.5 y 16 mmHg).^22^^,^^25^ No se ha tenido ningún caso de comunicación interventricular ni insuficiencia aórtica iatrogénica, debido al respeto de los límites quirúrgicos ya descritos. Las cifras de mortalidad hospitalaria de la miectomía en centros de gran experiencia es menor al 2%, el riesgo puede ser mayor en paciente ancianos y en aquellos con comorbilidades existentes.^26^^,^^27^

La mayor limitación del presente estudio es el número pequeño de pacientes sometidos a cirugía y las dificultades en el seguimiento a largo plazo por ser nuestro instituto un centro de referencia nacional y debido a que el paciente postoperado, una vez resuelta la patología quirúrgica, retorna a su hospital de origen.

## Conclusión

La miectomía septal extendida en pacientes con MHO, corrige la obstrucción del tracto de salida del VI, la IM y se observa una mejoría en la capacidad funcional. El tratamiento debe abarcar el componente miocárdico y valvular. Los resultados obtenidos en el presente estudio muestran que en nuestro instituto el tratamiento quirúrgico de la MHO es adecuado y se encuentra dentro de los parámetros internacionales.

## Financiación:

Ninguna
